# SARS-CoV-2, COVID-19 and Neurodegeneration

**DOI:** 10.3390/brainsci12070897

**Published:** 2022-07-08

**Authors:** Lars Tönges, Stephan Klebe

**Affiliations:** 1 Department of Neurology, Ruhr University Bochum, 44791 Bochum, Germany; 2 Department of Neurology, Essen University Hospital, 45147 Essen, Germany; stephan.klebe@uk-essen.de

The COVID-19 pandemic continues to affect many areas of our daily life. In addition to the immense societal and economic changes, medical care of patients has now been modified both in the outpatient and inpatient clinical settings. Neurological patients have experienced a drastic disruption in the provision of care, which was not similarly observed in the last few decades.

These profound changes also apply to Parkinson’s disease and other neurodegenerative disorders, including atypical Parkinson’s syndromes, Alzheimer’s and other dementias, ataxias, Huntington’s disease or dystonia syndromes. The diagnosis, implementation of outpatient or inpatient therapies and the initiation of new pharmacological or non-pharmacological treatment measures have changed considerably. However, the need to adapt our services for patients with neurodegenerative disease has also resulted in improvements that will remain highly valued also after the COVID-19 pandemic.

In this Special Issue, we present important findings on COVID-19, Parkinson’s and other neurodegenerative diseases in a series of publications. Fründt et al. studied the impact of the COVID-19 pandemic on the health care situation of people with Parkinson’s disease in Germany in the Care4PD study [1]. In a nationwide cross-sectional survey (ParCoPa), Wolff et al. examined the impact of the COVID-19 pandemic on Parkinson’s disease patient population from the perspective of the treating physicians in Germany [2]. In a study from Poland, Krzyston et al. performed an online survey to analyze the secondary impact of the COVID-19 pandemic on Parkinson´s patients with a focus on the level of activity, quality of life and PD-related symptoms [3]. A cross-sectional assessment of hospital admissions for neurodegenerative diseases (PSP, MSA, HD) during the first wave of the COVID-19 pandemic in Germany by Scherbaum et al. presents the nationwide care utilization for the first time [4]. 

The potential pathophysiological or etiological impact of SARS-CoV-2 on PD is discussed in four review articles. Morowitz et al. present a narrative review about the role of SARS-CoV-2 in modifying neurodegenerative processes in PD [5]. Drelich-Zbroja attempt to collate the existing scientific evidence regarding the possible role of SARS-CoV-2 in the pathophysiology of PD [6]. Krey et al. discuss whether a SARS-CoV-2 infection can lead to neurodegeneration and PD [7]. Goerttler et al. seek to clarify issues with SARS-CoV-2, COVID-19 and Parkinson’s disease in a critical review on the topic with a focus on post-COVID-19 syndrome [8]. 

The Special Issue is concluded by two articles about Alzheimer´s disease and ALS. The effects of a phone-based psychological intervention on the caregivers of patients with early onset Alzheimer’s disease (EOAD) are analyzed in a six-month study in Italy by De Stefano et al. [9]. De Marchi et al. describe an accelerated early progression of ALS over the COVID-19 pandemic in a tertiary expert ALS center in Italy [10]. 

In conclusion, the currently available data do not allow establishing a definitive etiological connection between COVID-19 and any of the neurodegenerative diseases listed above. It is foreseeable that the COVID-19 pandemic will occupy the scientific community for decades. Future studies and models will try to investigate the influence of COVID-19 on neurodegenerative diseases. The present Special Issue powerfully demonstrates the interplay and importance between the medical and social consequences of the COVID-19 pandemic for neurodegenerative diseases. 
